# The Anatomy of Suicide

**Published:** 1840

**Authors:** 


The Anatomy of Suicide. By *Forbes Winslow*, M.R.C.S. Author of "Physic and Physicians." Octavo, pp. 339. Renshaw, London. 1840.

The name of Winslow has long been honorably associated with the word "Anatomy"—but not the "Anatomy of Suicide." A non-medical friend of ours took up the volume from our table and seemed surprized at the word "Anatomy;" but we soon convinced him of its propriety, by handing to him Burton's "Anatomy of Melancholy." And indeed the book before us often reminded us of old Burton, by the variety and extent of the author's researches, and the amusing manner in which he has contrived to handle a most triste and repulsive subject. The nucleus of the volume formed a paper read at the Westminster Medical Society, and there excited great interest and protracted discussion. The subject is now developed to a very great extent, and illustrated by examples culled from all periods of history, ancient and modern. It is clearly the object of the author to prove, or at least render extremely probable, that *all* acts of suicide are committed under the influence of temporary insanity, partial or general. In this case, as in many others, the dispute is caused by the different definitions or interpretations of particular words. Thus, if every obliquity of intellect from the normal medium—every eccentricity of opinion—every crime against nature—every deviation from moral rectitude—every false notion entertained of our duty to God and man, be considered as grades or shades of insanity, why the door is thrown open enough to include suicide amongst the rest. But if we restrict the definition of insanity within the legal limits and the general acceptation of the word, we shall have great difficulty in bringing every case of suicide within the range of insanity. There is no doubt but that the more charitable view of the crime is to attribute it to temporary obscuration of reason; and this seems to be the impression of coroners' juries generally; but in treating the subject philosophically and medically, we must not sacrifice truth and justice at the shrine of mercy.

It would require half the Journal to notice half of the examples, histories, illustrations, and records of suicide compressed into Mr. Winslow's volume; and, as we are sure it will have a wide circulation, we shall be brief in our review. The work is divided into sixteen chapters, and we shall first glance at the eleventh—"Is the act of suicide the result of insanity?" The author justly observes that no law of Nature is more universal than that of self-preservation. Yet how often do we see men pursuing courses which they are convinced must entail sufferings or even death. A passage is quoted by Mr. Winslow from Dr. Rowley, which contains as complete a piece of special pleading, as we can remember to have seen.

"'Pain is an evil; death, the deprivation of every hope or comfort in this life. No man in his senses will burn, drown, or stab himself; for these all produce what are called evils; neither can any of these actions be executed without the probability of pain in the convulsive action or struggles of death. As no rational being will voluntarily give himself pain, or deprive himself of life, which certainly, while human beings preserve their senses, must be acknowledged evils, *it follows that every one who commits suicide is indubitably* non compos mentis, *not able to reason justly*, but is under the influence of false images of the mind; and therefore suicide *should ever be considered an act of insanity*.' "—222.

Thus, then, every man who is "not able to reason justly," is insane! Why there is not one man in every ninety-nine, who is "able to reason justly." We now see that, with such definitions of insanity, an ingenious writer or special pleader may easily prove that every case of self-destruction shews the individual to have reasoned "*unjustly*," and consequently to have been labouring under mental aberration. The illustrations which Mr. Winslow adduces of the natural instinct with which man clings to life, are apt and striking. It is well known that Samuel Johnson had the most unconquerable horror of death; and we strongly suspect that his belief in Christianity was very weak; else how could a man be so dreadfully apprehensive of passing from mortality to immortality? The Duke of Montebello, when lying in the jaws of Death, implored Napoleon to save him, when the surgeon was unable to stanch the hæmorrhage. This argued superstition quite as much as love of life. He must have been an addle-headed general to believe that Napoleon had the power of arresting the hand of death!

The following passage will shew the opinion of Mr. Winslow on the question at the head of this chapter.

"Is the act of suicide an evidence of mental derangement? Before this question can be satisfactorily answered, it would be necessary for us to consider that vexata quæstio—what is insanity? Have we an unfailing standard to which to appeal; an infallible *test* by which we can ascertain, with anything like a proximity to truth, the sanity of any mind? Perhaps, if we were to assert that we considered it impossible to point out the line of demarcation which separates the confines of a sane and insane condition of the mind, we might lay ourselves open to an attack. Again, were we bold enough to proclaim our non-adherence to what is considered as the orthodox faith in this matter, and assert that we viewed every departure from a healthy tone of mind, whether in its intellectual or moral manifestations, as an evidence of insanity, we might still more expose ourselves to the merciless lash of the critic, yet these are the opinions to which we should feel most disposed to give our assent. We must make a marked distinction between insanity considered as a *legal* and as a *medical* question; and it is greatly owing to our not keeping this essential difference in mind that so much useless reasoning and vituperation has arisen." 227.

And Again:—

"If we examine attentively the majority of cases of suicide, we shall find that the unfortunate persons have laboured, either for some time previously or at the very moment, under depression of spirits, anxiety of mind, and other symptoms of cerebral derangement. Very few cases of suicide take place in which you cannot trace the existence of previous mental depression, produced either by physical or moral agents. It may be said that lowness of spirits is not insanity; certainly not, according to the *legal* definition of the term; but we may always be assured, that if mental anxiety or perturbation be more than commensurate with the exciting cause, it may be presumed that the individual is labouring under the incipient indications of insanity." 229.

Sir W. Ellis, indeed, expressly declares that "lowness of spirits, ought to be regarded and treated as insanity." In the course of a long experience we do not remember to have ever treated a case where the patient did not complain, at one time or other, of "lowness of spirits"—hence, our practice has been exclusively among the insane, and we have every right to be admitted among the fraternity of mad doctors.

Mr. Winslow has certainly overturned, or greatly weakened some of the arguments adduced against his doctrine at the Westminster Medical Society.

"Was Cato perfectly sane when he sacrificed his life? We are disposed to think not. His whole conduct immediately preceding the last fatal act of his life evinces the extreme mental agitation under which he laboured; despair had taken possession of his faculties; the ambition and the hopes of years were prostrated in a moment to the dust, and to escape from a long life of tyranny, he perished on his own sword." 241.

The above argument, however, is far from being so strong as those urged in the cases of Colton, Romilly, and Castlereagh. It appears that Colton's life was embittered latterly by mental and physical sufferings, besides being involved in pecuniary difficulties. "His biographer states, that there was no doubt of Colton's insanity at the time of his death." In respect to Sir Samuel Romilly, we freely admit that the attempt which he made to restrain the hæmorrhage, after a certain quantity of blood had flowed, was a strong proof that he had laboured under cerebral congestion before the sad act took place. As for poor Lord Castlereagh, no man ever dreamt of his sanity of mind, even according to the limited and legal definition of insanity.

There is little doubt that the detection of the forgery of Rowley's poems, deranged the mind of poor Chatterton. At the time of his death he was in want of the common necessaries of life, realizing the affecting picture of the poet:—

"Homeless, near a thousand homes he stood,
And near a thousand tables pined and wanted food."

"In forming an estimate of the condition of a person's mind who has committed suicide, the coroner and jury should make particular inquiries into the following points:—First, as to the state of mind for some time prior to the act. In many, and in fact, in all cases, if proper evidence can be obtained, it will be discovered that the person has laboured under depression of spirits, either resulting from physical or mental causes. Inquiry should be instituted as to the presence of any disease of the stomach or liver which may have operated injuriously on the mind. In many cases it will be found that the suicide has received at some period of his life a blow on his head, giving rise to cerebral injury, which may remain latent for a great length of time, and suddenly manifest itself. Is insanity, particularly suicidal insanity, in the family? What was the person's natural character? Was he liable to sudden bursts of passion? Had his mind been dwelling on the subject of suicide? Was he monomaniacal, or remarkable for any peculiar eccentricity? All these various but important questions should be carefully sifted, should the coroner entertain any doubts as to the presence of mental derangement in such cases." 245.

We are quite ready to admit the propriety of all these precautions, without, however, subscribing to the doctrine that suicide is always the result of insanity, in the common sense of the word.

Reverting back to the first chapter of the work, we agree with our author that the examples of antiquity form no excuse for the act of suicide in modern times. The ancients considered death an eternal sleep, and consequently that we were not answerable in another world for acts done in the flesh. The Christian doctrine is different in all respects.

"The famous suicides of antiquity generally resulted from one of three causes:—First, it was practised by those who wished to avoid pain and personal suffering of body and mind; secondly, when a person considered the act as a necessary vindication of his honour; and thirdly, when life was sacrificed as an example to others." 2.

The first class, he thinks, is the most excusable. Pain, whether mental or corporeal, is a severe test of man's courage. Many persons have destroyed themselves to avoid falling into the hands of the enemy. The wife of Astrabal, and the celebrated Cleopatra are eminent examples. Demosthenes, Hannibal, Mithridates, and thousands of the ancients, put periods to their lives, and were applauded for the horrid act. Mr. Winslow enters into a long dissertation to prove that Cato was insane—that is, labouring under anxiety of mind, wounded pride, and lowness of spirits, when he stabbed himself. This we grant—provided Mr. W. admits that we are all insane at times.

"Two of the most distinguished men of antiquity who sacrificed their own lives were Brutus and Cassius. Before their battle with Cæsar on the plains of Philippi, these two warriors had a conversation on suicide. Cassius asked Brutus what his opinions were on the subject of self-destruction, provided fortune did not favour them in the contest in which they were about to be engaged. Brutus replied, that formerly he had embraced such sentiments as induced him to condemn Cato for killing himself; he deemed it an act of irreverence towards the gods, and that it was no evidence of courage. But, he continues, 'Now, in the midst of dangers, I am quite of another mind.' He then proceeds to tell Cassius of his determination to surrender up his life 'on the Ides of March.' He states no particular reasons for having changed his opinions on the subject of suicide. The issue of the battle is well known. Many things conspired to damp the courage of Cassius and Brutus. In imitation of Cæsar, Brutus made a public lustration for his army in the field, and during the ceremony an unlucky omen is said to have happened to Cassius. The garland he was to wear at the sacrifice was given to him the wrong side outwards; the person also who bore the golden image before Cassius stumbled, and the image fell to the ground. Several birds of prey hovered about his camp, and swarms of bees were seen within the trenches. Cassius, believing in the Epicurean philosophy, considered all these circumstances as disheartening omens of his fate. After the defeat of Cassius, he ordered his freedman to kill him, which he did by severing his head from his body.

Plutarch makes Brutus die most stoically. After having taken an affectionate leave of his friends, and having assured them that he was only angry with fortune for his country's sake, since he esteemed himself in his death more happy than his conquerors, he advised them to provide for their own safety. He then retired, and with the assistance of Strato, he ran his sword through his body." 11.

If this was madness "there was method in it." Petronius, the gay "*arbiter elegantiarum*," went to work as coolly as most of the ancients. He determined to indulge in a luxurious refinement of that death which he was preparing to encounter. He opened and closed his veins at pleasure—

slept during the intervals—or sauntered about and enjoyed the pleasures of conversation with his friends! Certes this example does not much strengthen the doctrine that suicide is always caused by insanity. Empedocles, the celebrated philosopher and poet, chose the crater of Etna for his tomb, and threw himself into it. We must close this seductive and amusing, though melancholy chapter, with the following extract.

"Ancient history affords us many noble examples of individuals who preferred voluntary death to dishonour and loss of character. If ever self-murder could be considered as in the slightest degree justifiable, it would be under such circumstances. Who cannot but honour the conduct of the noble virgins of Macedon, who threw themselves into the wells, and courted death, sooner than submit to the dishonourable proposals of the Roman governor! When Theoxena was pursued by the emissaries of Philip, king of Macedon, who had been guilty of murdering her first husband, she produced a dagger and a box of poison, and placing them before the crew of the ship in which she was endeavouring to make her escape, she said, 'Death is now our only remedy and means of vengeance; let each take the method that best pleases himself of avoiding the tyrant's pride, cruelty, and lust. Come on, my brave companions and family, seize the sword or drink of the cup, as you prefer an instantaneous or gradual death.' Some fell on the sword, others drank the poison until death was effected. After Theoxena had accomplished her designs, she threw herself into the arms of her husband, and they both plunged into the sea.

There can be no doubt that the doctrines of the ancient philosophers, especially the stoics, greatly increased the practice of suicide. "A wise man," says Diog. Laertius, "will quit life when oppressed with severe pains, or when deprived of any of his senses, or when labouring under desperate diseases." SENECA defended suicide. "Does life please you? Live on. Does it not? Go from whence you came." No vast wound is necessary. A mere puncture will secure your liberty! "You say it is a bad thing to be under the necessity of living;—but there is no necessity in the case. Thanks to the gods, nobody can be compelled to live." These were the principles of the wise Seneca!

In the second chapter, Mr. W. examines modern apologies for suicide—especially the writings of Hume, Donne, Rousseau, De Stael, Montesquieu, Gibbon, Voltaire, &c. However these writers may disguise their sentiments, they all disbelieved in a future state of existence. Our own opinion is that no man ever advocated or committed suicide, who was not either a materialist or a madman. We see no reason why the materialist may not, and in his proper senses too, weigh carefully the pros and cons, as to life and death, and if he find the balance in favour of the latter, draw the razor across his throat, and thus rid himself of all his evils.

Mr. Winslow criticises keenly the writings of the above authors in favour of suicide, and shews their fallacy or sophistry. We think it needless, after what we have just said, to scan the doctrines of these celebrated sceptics.

The more we examine Mr. Winslow's work, the more we are convinced of the impossibility of giving any thing like an analysis of it, so multitudinous are the topics, so elaborate the researches, and so terse the language. We can only dwell for a few minutes more on the last chapter—"Can SUICIDE be PREVENTED by LEGISLATIVE ENACTMENTS?

"The only legitimate object for which punishment can be inflicted is the prevention of crime. 'Am I to be hanged for stealing a sheep?' said a criminal at the Old Bailey, addressing the bench. 'No,' replied the judge; 'you are not to be hanged for stealing a sheep, but *that sheep may not be stolen.*' Every punishment, argues Beccaria, which does not arise from absolute necessity is unjust. There should be a fixed proportion between crimes and punishments. Crimes are only to be estimated by the injury done to society; and the end of punishment is, to prevent the criminal from doing further injury, as well as to induce others from committing similar offences.

The act of suicide ought not to be considered as a crime in the legal definition of the term. It is not an offence that can be deemed cognizable by the civil magistrate. It is to be considered a sinful and vicious action. To punish suicide as a crime is to commit a solecism in legislation. The unfortunate individual, by the very act, of suicide, places himself beyond the vengeance of the law; he has anticipated its operation; he has rendered himself amenable to the highest tribunal—viz. that of his Creator; no penal enactments, however stringent, can affect him. What is the operation of the law under these circumstances? A verdict of *felo-de-se* is returned, and the innocent relations of the suicide are disgraced and branded with infamy, and that too on evidence of an *ex-parte* nature. It is unjust, inhuman, unnatural, and unchristian, that the law should punish the innocent family of the man who, in a moment of frenzy, terminates his own miserable existence. It was clearly established, that before the alteration in the law respecting suicide, the fear of being buried in a cross-road, and having a stake driven through the body, had no beneficial effect in decreasing the number of suicides; and the verdict of *felo-de-se*, now occasionally returned, is productive of no advantage whatever, and only injures the surviving relatives." 335.

It is a barbarous law no doubt, and is now nearly obsolete. But it is not the only barbarous law on our statute book. The man attainted with high treason forfeits his life, and his relatives are punished, after his death, by the forfeiture of his property, which ought to go to his heirs who have committed no crime. This is a still harder case than the other: for we much doubt whether the surviving relatives would not prefer a verdict of *felo-de-se* to that of *insanity*, than which is nothing more dreaded among families—the stain being a *physical* one, and considered, whether true or false, to be capable of extension by hereditary descent, which is not the case with a moral stain. The whole turns on the question—do these penalties and forfeitures act as checks on the commission of suicide and treason? In the *latter* case, it is probable that they do—in the *former*, we think they are almost entirely inefficacious. The following quotation will shew that solemn JUDGES on the Bench have considered lunacy as nearly coeval with human nature, and the inevitable lot of every human being, at one or other time of life!

"If the mind be overpowered by 'grief, sickness, infirmity, or *other accident*,' as Sir Mathew Hale expresses it, the law presumes the existence of lunacy." 336.

Having shewn, then, that no penal law can prevent suicide, our author asks, is there no other means of staying the progress of the Act?

"In the prevention of suicide, too much stress cannot be laid on the importance of adopting a well-regulated, enlarged, and philosophic system of education, by which all the *moral* as well as the intellectual faculties will be expanded and disciplined. The education of the intellect without any reference to the moral feelings is a species of instruction calculated to do an immense amount of injury. The tuition that addresses itself exclusively to the perceptive and reflective faculties is not the kind of education that will elevate the moral character of a people. Religion must be made the basis of all secular knowledge. We must be led to believe that the education which fits the possessor for another world is vastly superior to that which has relation only to the concerns of this life. We are no opponents to the diffusion of knowledge; but we are to that description of information which has only reference 'to the life that is, and not to that which is to be.' Such a system of instruction is of necessity defective, because it is partial in its operation. Teach a man his duty to God, as well as his obligations to his fellow-men; lead him to believe that his life is not his own; that disappointment and misery is the penalty of Adam's transgression, and one from which there is no hope of escaping; and, above all, inculcate a resignation to the decrees of Divine Providence. When life becomes a burden, when the mind is sinking under the weight of accumulated misfortunes, and no gleam of hope penetrates through the vista of futurity to gladden the heart, the intellect says, 'Commit suicide, and escape from a world of wretchedness and woe;' the moral principle says, 'Live; it is your duty to bear with resignation the afflictions that overwhelm you; let the moral influence of your example be reflected in the characters of those by whom you are surrounded.'" 338.

From a short chapter on the appearances after death in suicide, we make the following extract in conclusion.

" From an examination of the particulars of 1333 cases of persons who have committed suicide, and who have been examined after death, the following analysis is made. The particulars of the cases referred to are recorded in the works of Pinel, Esquirol, Falret, Foderé, Arntzenius, Schlegel, Burrows, Haslam, &c.

| | |
|---|---|
| Thickness of cranium .. .. .. .. .. | 150 |
| No apparent structural change .. .. | 100 |
| Bony excrescences .. .. .. .. .. .. | 50 |
| Tumours in brain .. .. .. .. .. .. | 10 |
| Simple congestion .. .. .. .. .. .. | 300 |
| Disease of membranes .. .. .. .. .. | 170 |
| Disease of lungs .. .. .. .. .. .. | 100 |
| Softening of brain .. .. .. .. .. .. | 100 |
| Appearances of inflammation in brain .. | 90 |
| Disease of stomach .. .. .. .. .. .. | 100 |
| Disease of intestines .. .. .. .. .. | 50 |
| Disease of liver .. .. .. .. .. .. | 80 |
| Suppressed natural secretions .. .. | 15 |
| Disease of heart .. .. .. .. .. .. | 10 |
| Syphilitic disease .. .. .. .. .. .. | 8 |
| | 1333. |

Accretions of the membranes of the brain are often found in suicides. The dura mater is often ossified, and the pia mater inflamed, and the arachnoid thickened. Osiander considers congestion of the vessels of the brain a frequent cause of suicide." 281.

Here we must close our very imperfect notice of a volume which does infinite credit to the head and the heart of the author.

